# A novel matrix dispersion based on phospholipid complex for improving oral bioavailability of baicalein: preparation, *in vitro* and *in vivo* evaluations

**DOI:** 10.1080/10717544.2017.1311968

**Published:** 2017-04-24

**Authors:** Yang Zhou, Wujun Dong, Jun Ye, Huazhen Hao, Junzhuo Zhou, Renyun Wang, Yuling Liu

**Affiliations:** 1 State Key Laboratory of Bioactive Substance and Function of Natural Medicines and; 2 Beijing Key Laboratory of Drug Delivery Technology and Novel Formulation, Institute of Materia Medica, Chinese Academy of Medical Sciences & Peking Union Medical College, Beijing, PR China

**Keywords:** Matrix dispersion, baicalein, phospholipid complex, oral bioavailability, dissolution

## Abstract

Phospholipid complex is one of the most successful approaches for enhancing oral bioavailability of poorly absorbed plant constituents. But the sticky property of phospholipids results in an unsatisfactory dissolution of drugs. In this study, a matrix dispersion of baicalein based on phospholipid complex (BaPC-MD) was first prepared by a discontinuous solvent evaporation method, in which polyvinylpyrrolidone-K30 (PVP-K30) was employed for improving the dispersibility of baicalein phospholipid complex (BaPC) and increasing dissolution of baicalein. The combination ratio of baicalein and phospholipids in BaPC-MD was 99.39% and baicalein was still in a complete complex state with phospholipid in BaPC-MD. Differential scanning calorimetry (DSC), X-ray diffraction (XRD), scanning electron microscopy (SEM) and Fourier Transform Infrared (FTIR) analyzes demonstrated that baicalein was fully transformed to an amorphous state in BaPC-MD and phospholipid complex formed. The water-solubility and *n*-octanol solubility of baicalein in BaPC-MD significantly increased compared with those of pure baicalein. Compared with baicalein and BaPC, the cumulative dissolution of BaPC-MD at 120 min increased 2.77- and 1.23-fold, respectively. *In vitro* permeability study in Caco-2 cells indicated that the permeability of BaPC-MD was remarkably higher than those of baicalein and BaPC. Pharmacokinetic study showed that the average *C*_max_ of BaPC-MD was significantly increased compared to baicalein and BaPC. AUC_0–14 h_ of BaPC-MD was 5.01- and 1.91-fold of baicalein and BaPC, respectively. The novel BaPC-MD significantly enhanced the oral bioavailability of baicalein by improving the dissolution and permeability of baicalein without destroying the complexation state of baicalein and phospholipids. The current drug delivery system provided an optimal strategy to significantly enhance oral bioavailability for poorly water-soluble drugs.

## Introduction

The oral administration of drugs is the most common route because of its good patient compliance and the lower cost of manufacturing compared with other routes. However, low bioavailability is a major challenge for the oral dosage form development. The poor bioavailability of drugs is mainly attributed to the poor aqueous/lipid solubility and low plasma membrane permeability (Lipinski et al., 2001). Therefore, suitable formulation strategy to enhance the oral drug absorption is a topic of great interest in pharmaceutical study.

Among the numerous approaches explored in recent years for the improvement of oral bioavailability of poorly absorbed drugs, phospholipid complex has received increasing attention and become one of the most successful methods (Khan et al., 2013). Phospholipid complex exhibits the significant capability to increase the water-solubility of drugs and also promote the permeation across the intestinal epithelium in most studies (Yue et al., 2010; Singh et al., 2014). However, due to the sticky nature of phospholipids, phospholipid complex tends to aggregate and agglomerate. This disadvantage results in an unsatisfactory dissolution and oral absorption of drugs, although phospholipid complex could increase the dissolution of drugs to a certain extent compared with free drugs. Therefore, it is necessary to choose suitable carriers to disperse phospholipid complex and thereby to increase the dissolution of drugs.

Recently several phospholipid-complex based drug delivery systems have been developed to improve the dispersibility of phospholipid complex for enhancing the dissolution and oral bioavailability of drugs, such as solid dispersion of berberine–phospholipid complex/TPGS 1000/SiO_2_ and a solidified phospholipid complex (Zhang et al., 2014; Jiang et al., 2016). But there was no report about the combination ratio of drug and phospholipid in these studies. The combination ratio is usually employed as an important index for the formation of phospholipid complex. A number of studies have shown that phospholipid complex with high combination ratio could significantly enhance the oral bioavailability of drugs compared with the physical mixture of drugs and phospholipids (Yue et al., 2010; Li et al., 2015). This demonstrates that complex state between drugs and phospholipids is a crucial factor for the enhancement of oral bioavailability by phospholipid complex technique.

The drug used in the present study was baicalein, isolated from the root of *Scutellaria baicalensis* (Yang et al., 2000). Baicalein has a variety of pharmacological effects, including anti-inflammatory activity (Luo et al., 2016), antioxidative effects (Shao et al., 2002), anti-allergenic activity (Kimata et al., 2000), antiviral effects (Evers et al., 2005; Chen et al., 2011), anticancer effects (Wang et al., 2010; Zheng et al., 2014) and neuroprotective effects (Cheng et al., 2008; Moon & Park, 2015). Due to its poor aqueous solubility, the oral bioavailability of baicalein is too low to satisfy the need of clinical treatment. Although several formulation approaches have been reported to enhance the oral absorption of baicalein, such as HP-β-CD co-lyophilized product (Liu et al., 2006), nanocrystal (Zhang et al., 2011), self-microemulsifying drug delivery system (Liu et al., 2012), long-circulating nanoliposomes (Liang et al., 2013) and solid dispersion (Yan et al., 2008; Zhang et al., 2014), there is still space for other formulation approaches. In the preliminary study, we have successfully prepared baicalein-phoapholipid complex (BaPC) that could enhance the oral bioavailability of baicalein in rats. However, it was also found that BaPC was too sticky with low dispersibility, indicating that there is still some room for further improvement in the dissolution of baicalein. Thus, some effective ways need to be adopted to overcome this limitation.

In the present study, for the purpose of further enhancing the dissolution and oral bioavailability of baicalein based on phospholipid complex without destroying complexation state of baicalein and phospholipids, a novel matrix dispersion of baicalein based on phospholipid complex (BaPC-MD) was first prepared by the discontinuous solvent evaporation method. Hydrophilic polyvinylpyrrolidone-K30 (PVP-K30), which is a high molecular polymer and usually applied for improving the dissolution of drugs, was used as a dissolution enhancer to overcome the shortcoming of easy aggregation of phospholipid complex (Sharma & Jain, 2010; Cong et al., 2014). The combination ratio of drug with phospholipid was firstly employed to confirm the complex state between baicalein and phospholipids in BaPC-MD. BaPC-MD was characterized by differential scanning calorimetry (DSC), X-ray diffraction (XRD) and scanning electron microscopy (SEM). Fourier Transform Infrared (FTIR) was used to confirm the formation of BaPC. Solubility in water and *n*-octanol and dissolution profiles of pure baicalein, BaPC and BaPC-MD were evaluated and compared. In addition, we compared the oral bioavailability and *C*_max_ of pure baicalein, BaPC and BaPC-MD in SD rats.

## Materials and methods

### Materials

Baicalein (purity 98%) was purchased from Nanjing Tingze Medical Science and Technology Development Co. Ltd. (Nanjing, China). Soybean phospholipids (S75) were purchased from Lipoid GmbH (Ludwigshafen, Germany), and the phosphatidylcholine content was approximately 75% (w/w). PVP-K30 was supplied by Chemical Reagents (Beijing, China). Baicalein and baicalin reference substances were purchased from the National Institute for the Control of Pharmaceutical and Biological Products (Beijing, China). High-performance liquid chromatography (HPLC)-grade methanol and acetonitrile were obtained from Sigma (Sigma-Aldrich, Shanghai, People’s Republic of China). Modified Eagle’s medium (MEM), fetal bovine serum (FBS) and all other reagents were obtained from Gibco Laboratories (Life Technologies Inc., Grand Island, NY, USA). Transwell™ plates of 12 wells (12 mm, polycarbonate filter, pore size 0.4 μm) were purchased from Corning Costar (Cambridge, MA). All other reagents were analytical grade.

### Preparation of BaPC-MD

BaPC-MD was prepared at a baicalein: phospholipid: PVP-K30 ratio of 1:3.5:2 (w/w/w) by a discontinuous solvent evaporation method. Briefly, baicalein and phospholipids were accurately weighted into in a round-bottom flask and co-dissolved in tetrahydrofuran at room temperature. Subsequently, tetrahydrofuran was evaporated by rota-evaporator under vacuum at 40 °C, and PVP-K30 was added to the complex system when solid content was above 0.8 g/mL. The evaporation was continued until all the solvent was removed. The resultant complex was collected and vacuum dried, and flushed with nitrogen and stored at room temperature. BaPC was prepared with the similar procedure without PVP-K30.

### Determination of the combination ratio of baicalein and phospholipid

The combination ratio of baicalein was measured according to the difference in solubility among baicalein, BaPC-MD and BaPC. Both BaPC-MD and BaPC could be dissolved in n-hexane, but free baicalein or uncomplexed baicalein could not be dissolved in n-hexane and all could be dissolved in anhydrous ethanol. The combination ratio of baicalein and phospholipid was calculated according to the following equation:

(1) $$\text{\{\textbackslash{}rm Combination\textbackslash{} ratio\}}\left(\%\right)=W_{\text{\_\{\textbackslash{}rm comb\}}}/W_{\text{\_\{\textbackslash{}rm total\}}}\backslash times100$$

where *W*_comb_ is the combination amount of baicalein in the BaPC-MD or BaPC, *W*_total_ is the total amount of baicalein in the BaPC-MD or BaPC.

The content of baicalein was determined by HPLC method. About 10 μl aliquots of the resulting solution were injected into an HPLC system (Agilent1200, Agilent Technologies, Palo Alto, CA). The stationary phase, ODS C18 column (250 mm × 4.6 mm, 5 μm), was kept at room temperature. The mobile phase was a mixture of methanol: 0.05% H_3_PO_4_ (65:35, v/v). The flow rate was 1.0 mL/min and effluents were monitored at 275 nm.

### Characterization of BaPC-MD

#### Differential scanning calorimetry

Samples were sealed in an aluminum crimp cell and heated at the speed of 10 °C/min from 25 °C to 350 °C in atmospheric nitrogen (60 mL/min). The peak transition-onset temperatures of baicalein, BaPC, PM-BaPC/PVP, BaPC-MD and a physical mixture of baicalein, phospholipids and PVP-K30 were determined with an Exstar6000 differential scanning calorimeter (Seiko, Japan).

#### X-ray diffractometry

X-ray diffractograms (D8 advance, BRUKER, Germany) were scanned with a diffraction angle increasing from 3° to 60°, a 2*θ* angle with a steep angle of 0.02°, and a count time of 0.1 s per step.

#### Scanning electron microscopy

Baicalein, BaPC and BaPC-MD were each coated with platinum in a sputter coater, and their surface morphology was viewed and photographed with a scanning electron microscope (Hitachi S4800; Hitachi Ltd., Shiga, Japan).

#### Fourier transform infrared

The infrared spectra of pure baicalein, phospholipids, a physical mixture of baicalein and phospholipids and BaPC were obtained by IR Spectrometry (Nicolet 5700; Thermo Electron Scientific Instruments, Waltham, MA). The samples were taken in KBr pellets and scanned over the range 4000–400 cm^−1^.

### Solubility studies

Solubility determination of baicalein, BaPC, PM-BaPC/PVP and BaPC-MD was carried out by adding excess samples to water or *n*-octanol in sealed containers at 25 °C. The liquid samples were shaken and centrifuged at 14 000 rpm. The supernatant was filtered, and the filtrate was diluted with an appropriate amount of anhydrous ethyl alcohol for the measure of baicalein by HPLC method as previously described. Each experiment was performed in triplicate.

### Dissolution studies

The *in vitro* dissolution studies were carried out using the paddle method according to Chinese pharmacopeia (2015 edition). 0.5% SDS was used as the dissolution medium. The paddle speed was set to 75 rpm and the water bath temperature was maintained at 37 ± 0.5 °C. Baicalein, BaPC, PM-BaPC/PVP or BaPC-MD (equivalent to 100 mg of baicalein) was added into the stirred dissolution medium at beginning of the study, respectively. 2.5 mL dissolution medium were withdrawn at different time intervals and an equal volume of fresh medium was added to each flask. The withdrawn samples were then filtered using a 0.45 μm membrane and analyzed by HPLC at a wavelength of 275 nm. Each experiment was performed in triplicate.

### In vitro *permeability studies*

The human colon adenocarcinoma cell line Caco-2, used as a model system for studying gastrointestinal epithelial permeability, was purchased from the Cell Resource Center, Chinese Academy of Medical Sciences (Beijing, China). Caco-2 cells were cultured in MEM medium supplemented with 10% (v/v) FBS, 1% (v/v) NEAA, penicillin (100 U/mL) and streptomycin (100 μg/mL) and cultured in a humidified incubator with 5% CO_2_, at 37 °C. All cells used in this study were between passages 36 and 40. Caco-2 cells were seeded at a density of about 6.25 × 10^4^ cells/cm^2^ in 12-wells Transwell® plates and cells were cultured for 21 days to reach confluence and differentiation. Only the wells of cell monolayer with transepithelial electrical resistance (TEER) value above 500 Ω cm^2^ were used for the transport assays.

The transport assay was performed in apical-basolateral direction. The test solutions of baicalein, BaPC and BaPC-MD at a dose equivalent to 10 μg/mL of baicalein were prepared by adding appropriate amount of each formulation to Hanks balanced salt solution (HBSS, pH 7.4) and dispersing completely by vortex. The Caco-2 cell monolayers were washed twice with prewarmed HBSS before the permeability studies. The experiments were initiated by adding 0.5 mL of test solution to the apical chamber (AP) and 1.5 mL of HBSS to the basolateral chamber. Plates were agitated on an orbital shaker at 50 rpm during the whole incubation period. Samples of 200 μL were then taken from basolateral chamber and immediately replaced with the same volume of 37 °C HBSS at predetermined time intervals 30, 60, 90, 120 min. Samples were then diluted with 0.2 mL of 0.5% ascorbic acid in methanol solution, and stored at −20 °C until analysis.

The concentration of baicalein in the samples of transport buffer was determined by HPLC analysis. The apparent permeability coefficient (Papp) across Caco-2 cell monolayer was calculated for permeability studies with the following equation:

(2) $$\mathrm{Papp}=\left(\frac{dQ}{dt}\right)\left(\frac{1}{{\mathrm{AC}}_{0}}\right)$$

where Papp is the apparent permeability coefficient (cm/s), *dQ* is the cumulative amount of baicalein across the Caco-2 barrier at time *dt, A* is the surface area of the Transwell® membrane (1.12 cm^2^), *C*_0_ is the initial concentration of baicalein in the apical side (μg/mL).

### In vivo pharmacokinetic study

#### Pharmacokinetic study of BaPC-MD

The *in vivo* pharmacokinetic study of baicalein, BaPC and BaPC-MD was performed in SD male rats. Eighteen male rats (body weight 180–220 g) were divided randomly into three groups. The rats were fasted for 12 h but allowed to take water freely. Various preparations at a dose equivalent to 75 mg/kg baicalein were orally administered to each group of rats.

#### Plasma sample preparation and validity

Approximately 0.5 mL blood sample was taken from the eyeground veins at predetermined time points. The plasma obtained after centrifugation (10 min, 4000 rpm) was stored at −20 °C until analyzed. When each plasma sample was thawed, 100 μL of plasma was spiked with 50 μL of 6-Hydroxyflavone (6-HF, 10 μg/mL) as the internal standard. 5 μL of ascorbic acid (200 mg/mL) and 300 μL of methanol were added. This mixture was agitated for 60 s and then centrifuged (10 min, 12 000 rpm). The supernatant was transferred into a clear, tapered centrifuging tube and evaporated under nitrogen at 40 °C.

The residues were resuspended in 100 μL of a methanol:water mixture (80:20, v/v), agitated for 30 s, and then centrifuged (5 min, 12 000 rpm). Aliquots (20 μL) of the supernatant were injected into an HPLC system (HP 1200 series; Agilent, USA). The stationary phase, an Agilent C18 column (250 mm × 4.6 mm, 5 μm), was kept at 25 °C. The mobile phase was composed of a mixture of 0.05% H_3_PO_4_ and acetonitrile that was run in a gradient elution program. The flow rate was 1.0 mL/min, and the effluent was monitored at 320 nm.

### Statistical analysis

The pharmacokinetic parameters were computed using the software program DAS 2.0. All values were expressed as the mean ± the standard deviation (SD). Statistical analysis was carried out using Student’s *t*-test. Differences were considered statistically significant at *p* < 0.05.

## Results and discussion

### Preparation and the combination ratio of complexes

Drug–phospholipid complex was prepared by forming the weak force such as hydrogen bond or van der Waals force between drug and phospholipid. And complexation conditions or additional carriers may affect the interaction between the drug and phospholipid or the combination ratio of phospholipid complex. Under the optimal conditions, the combination ratio of baicalein and phospholipid in the BaPC was 99.73 ± 0.92%, indicating that the baicalein did fully complex with phospholipid. Unfortunately, the phospholipid complex was sticky and difficult to be dispersed for further preparation.

Thus, we attempted to develop a novel dispersion to overcome these limits using PVP-K30 as a matrix carrier. PVP-K30 is a commonly used water-soluble and carbonyl-rich carrier for solid dispersion. But the carbonyl groups or hydroxyl on the surface of such carrier may have a great effect on the formation of phospholipid complex. Some results also showed the presence of hydrogen bonds between the carbonyl groups of PVP and the hydroxyl groups of flavonoids (Kanaze et al., 2010). After mixing baicalein, phospholipid and PVP-K30 in ethanol or other solvents, phospholipid complex based dispersion was prepared by the simple solvent evaporation or spray drying method, in which the complex state of baicalein and phospholipid was partially destroyed by PVP-K30 and the combination ratio was below 70%. Few studies have focused on this kind of phenomenon.

In this study, the combination ratio of BaPC-MD was 99.39 ± 0.54% (*n* = 3), indicating that baicalein was still in the complexation state in BaPC-MD prepared by the discontinuous solvent evaporation method. This enabled us to take full advantage of the permeation enhancement potential of BaPC. Meanwhile, BaPC-MD exhibited fine particles and good dispersity. The mechanism of the formation of BaPC-MD and the effects using other carrier materials as matrix need to be evaluated in our further studies.

### Characterization of BaPC-MD

#### DSC

**Figure 1.**
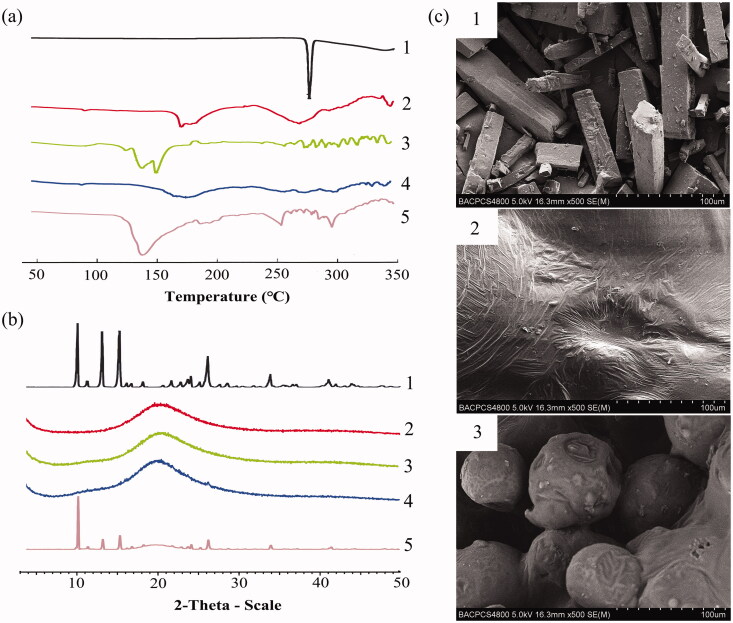
Characterization of BaPC-MD. (a) Differential scanning calorimetry ther-mograms of 1: pure baicalein; 2: BaPC; 3: PM-BaPC/PVP; 4: BaPC-MD; 5: a physical mixture of baicalein, phospholipids, and PVP-K30. (b) X-ray diffraction patterns of 1: pure baicalein; 2: BaPC; 3: PM-BaPC/PVP; 4: BaPC-MD; 5: a physical mixture of baicalein, phospholipids, and PVP-K30. (c) Scanning electron microscopic micrographs of 1: pure baicalein; 2: BaPC; 3: BaPC-MD.

To acquire the information about possible interactions between drugs and excipients, DSC thermograms of pure baicalein, BaPC, PM-BaPC/PVP, BaPC-MD and a physical mixture of baicalein, phospholipids, and PVP-K30 were studied in Figure 1(a). DSC thermogram of baicalein showed a distinct endothermic peak at 272.3 °C, which was indicative of its crystalline state. However, BaPC exhibited two small peaks that were different from that of baicalein and phospholipids, demonstrating an interaction between them. Likewise, the DSC curve of BaPC-MD showed that the endothermic peak of baicalein disappeared, indicating the amorphous state of baicalein in BaPC-MD. DSC analysis indicated that BaPC and BaPC-MD may generate a change in baicalein from a crystalline to an amorphous state.

#### XRD

The X-ray diffraction patterns of pure baicalein, BaPC, PM-BaPC/PVP, BaPC-MD and a physical mixture of baicalein, phospholipids, and PVP-K30 are shown in Figure 1(b). The X-ray spectrum of baicalein exhibited numerous sharp diffraction peaks, which were indicative of its highly crystalline structure. In contrast, there were no diffraction peaks of baicalein in BaPC, suggesting almost complete drug amorphization. Also, the XRD pattern of BaPC-MD showed that diffraction peaks of baicalein disappeared, which proved that baicalein in BaPC-MD existed in an amorphous state.

#### SEM

The surface morphologies of pure baicalein, BaPC and BaPC-MD are shown in Figure 1(c). The morphology of pure baicalein showed that it appeared in rod-like crystal state. However, BaPC consisting of drugs and phospholipids was block shaped and no baicalein crystals were observed, which indicated that baicalein was present in BaPC in an amorphous state. In addition to the absence of baicalein crystals, BaPC-MD appeared smaller spheres with the size of approximate 50 μm compared with BaPC. It was inferred that the solubility and dissolution of baicalein in BaPC-MD might be enhanced by the amorphous state of baicalein and increasing the surface area of BaPC.

#### FTIR

**Figure 2.**
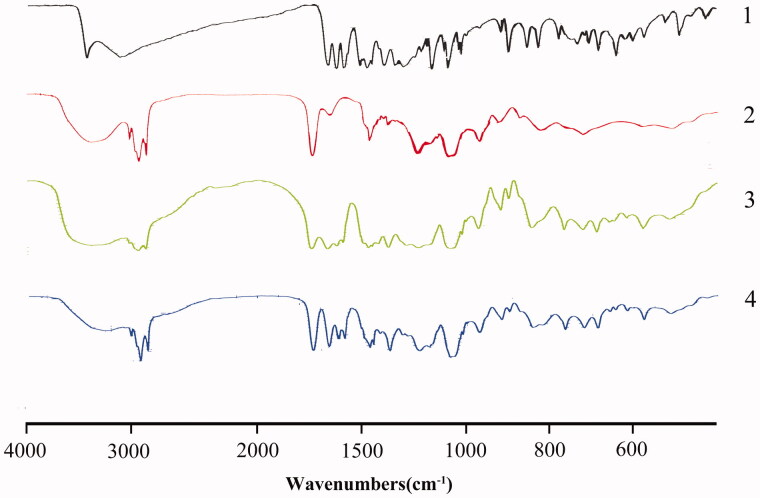
FTIR spectra of 1: pure baicalein; 2: phospholipids; 3: a physical mixture of baicalein and phospholipids; 4: BaPC.

Figure 2 shows the FTIR spectra of pure baicalein, phospholipids, a physical mixture of baicalein and phospholipids and BaPC. The baicalein spectrum exhibited characteristic peaks at 3408.6 cm^−1^ (O–H stretching) and 1657.7 cm^−1^ (C = O stretching). And the phospholipid spectrum exhibited characteristic peaks at 2925.7 cm^−1^ and 2853.8 cm^−1^ (C–H stretching band of long fatty acid chain), 1739.0 cm^−1^ (C = O stretching), 1237.7 cm^−1^ (P = O stretching), 1088.4 cm^−1^ (P–O–C stretching) and 970.2 cm^−1^ (N^+^(CH_3_)_3_ stretching). The spectrum of the physical mixture (1:3.5, w/w) showed a summation of the vibrational frequencies of the individual components. However, the characteristic absorption peak (C = O) of baicalein was shifted to higher wave number and the peak (O–H stretching) broadened in the spectrum of BaPC. In addition, the characteristic absorption peaks of the polar head of phospholipids moved from 1237.7 cm^−1^ (P = O stretching) to 1229.1 cm^−1^, yet the characteristic peaks at 2925.7 cm^−1^ and 2853.8 cm^−1^ (C–H stretching band of long fatty acid chain) of phospholipids were still found in the spectrum of BaPC. These results demonstrated that no new chemical bond was formed, but some weak physical interaction between baicalein and polar head of phospholipids, such as hydrogen bond or van der Waals forces (Yanyu et al., 2006; Ahmad et al., 2016), probably occurred to form BaPC.

### Solubility studies

Table 1 displays the solubility of pure baicalein, BaPC, PM-BaPC/PVP and BaPC-MD in water or *n*-octanol. The results showed that the aqueous solubility and lipid solubility of BaPC were both dramatically increased in comparison with those of pure baicalein. Moreover, BaPC-MD exhibited further significant enhancement in the aqueous solubility compared with free baicalein and BaPC. The amorphization of baicalein and the amphiphilic nature and solubilizing characteristics of phospholipids may likely explain this increase in the solubility of BaPC (Khan et al., 2013; Alexander et al., 2016; Kawakami, 2016). Consequently, the solubility of BaPC and BaPC-MD in both water and octanol were significantly increased compared with that of pure baicalein.

**Table 1.** Solubility of baicalein, BaPC, PM-BaPC/PVP and BaPC-MD in water or *n*-octanol at 25 °C (*n* = 3).

Samples	Solubility in water (μg/mL)	Solubility in *n*-octanol (mg/mL)
Baicalein	17.50 ± 0.08	3.14 ± 0.10
BaPC	970.36 ± 12.47*	32.27 ± 1.31*
PM-BaPC/PVP	1191.44 ± 4.78* #	30.47 ± 1.41*
BaPC-MD	1510.04 ± 15.14* #	30.97 ± 1.42*

Each value represented the mean ± SD.

* *p* < 0.05 versus baicalein.

# *p* < 0.05 versus BaPC.

### Dissolution studies

**Figure 3.**
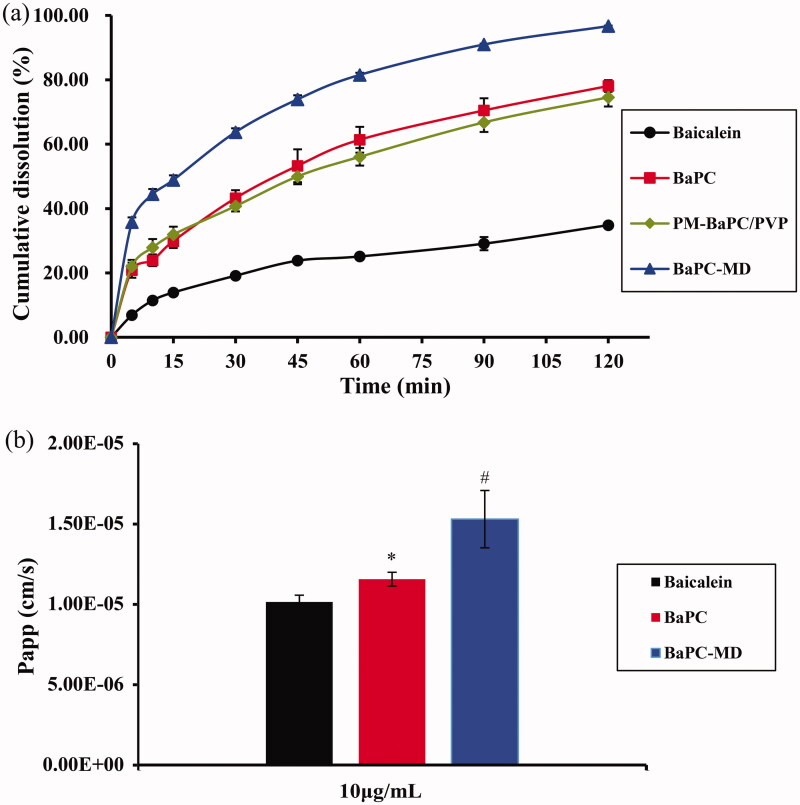
(a) Cumulative dissolution profiles of baicalein from 1: pure baicalein; 2: BaPC; 3: a physical mixture of BaPC and PVP-K30; 4: BaPC-MD in 0.5% SDS. (b) The apparent permeability coefficients of 1: baicalein; 2: BaPC; 3: BaPC-MD at an equivalent dose of 10 μg/mL baicalein through Caco-2 cell monolayers. Each value represented the mean ± SD (*n *=* *3).

Before oral drugs permeate the biological membranes of the gastrointestinal tract to reach the systemic circulation, they must firstly dissolve in gastrointestinal fluids. Drugs with poorly aqueous solubility exhibit low dissolution, leading to poor oral bioavailability. Therefore, enhancing the dissolution of poorly aqueous soluble drugs is a key problem in pharmaceutical research. Figure 3(a) shows the dissolution profile of baicalein from pure baicalein, BaPC, a physical mixture of BaPC and PVP-K30 and BaPC-MD in 0.5% SDS, respectively. The drug dissolution of MDC-BaPC was 35.75% within the first 5 min, while that of pure baicalein and BaPC was only about 6.88% and 20.84% within the same time frame, demonstrating that the dissolution rate of MDC-BaPC was higher than that of BaPC and pure baicalein. Compared with pure baicalein and BaPC, the cumulative dissolution of BaPC-MD at 120 min increased by 2.77-fold and 1.23-fold, respectively.

The dissolution of baicalein from BaPC-MD was significantly enhanced in comparison with pure baicalein, BaPC and a physical mixture of BaPC and PVP-K30. This could be attributed to several reasons as follows: firstly, PVP-K30 as a hydrophilic material with good wettability could improve the dispersibility of BaPC and thereby increase the surface area of BaPC; secondly, phospholipids possessed the solubilization and wetting properties, which contributed to the increased dissolution of baicalein; thirdly, an amorphous state of baicalein in BaPC-MD improved the aqueous solubility compared to the crystalline state (Wei et al., 2017).

### In vitro permeability studies in Caco-2 cell monolayers

The apparent permeability coefficients of baicalein, BaPC and BaPC-MD (at an equivalent dose of 10 μg/mL baicalein) at pH 7.4 were compared in Figure 3(b). The Papp values of baicalein in the presence of free baicalein, BaPC and BaPC-MD were 1.01 × 1^−5^, 1.16 × 1^−5^ and 1.53 × 1^−5 ^cm/s, respectively. These results demonstrated that BaPC and BaPC-MD significantly increased the permeability of baicalein through Caco-2 monolayer compared to the pure baicalein. The possible explanation for the results might be due to the amphiphilicity of phospholipids, which functioned as surfactants and assisted the drug to traverse across the membrane (Saoji et al., 2016). Moreover, the permeability of baicalein across Caco-2 monolayer significantly improved in the presence of BaPC-MD compared with BaPC. This result may be attributed to two aspects. One reason was that the complexation state of baicalein and phospholipid in BaPC-MD was not destroyed by PVP-K30, which guaranteed that BaPC fully promoted the penetration of baicalein across the bilayer lipid cell membrane. The other one was that the enhancement of dissolution rate from BaPC-MD might also facilitate the improvement of permeability of baicalein.

### Pharmacokinetic study in rats

Our preliminary study showed that baicalein was quickly metabolized to baicalin in plasma and was almost at the LLOD level at 30 min post-dosing. The clearance of the mainly conjugate baicalin was relatively slow, which was still detectable after 720 min. These results were consistent with the previous studies (Zhang et al., 2011). Baicalin is the main conjugated metabolite of baicalein in plasma that possesses the same pharmacological effects with baicalein. Therefore, we chose baicalin as the target for *in vivo* bioavailability studies and all pharmacokinetic parameters below were obtained based on the analysis of plasma concentration of baicalin.

An HPLC analytic method was established for the simultaneous determination of baicalin. Baicalin and 6-HF were separated from impurities in plasma extracts with retention times of 9.6 and 19.3 min, respectively. The calibration curve of baicalin was linear *Y* = 0.1257*X* + 0.0008 (*r* = 0.9992, where *X* is the concentration of baicalin, and *Y* is the corresponding peak area ratio baicalin/6-HF) in the concentration range of 0.05–50 μg/mL. The limit of quantification and the limit of detection were 50 ng/mL and 20 ng/mL, respectively.

**Figure 4.**
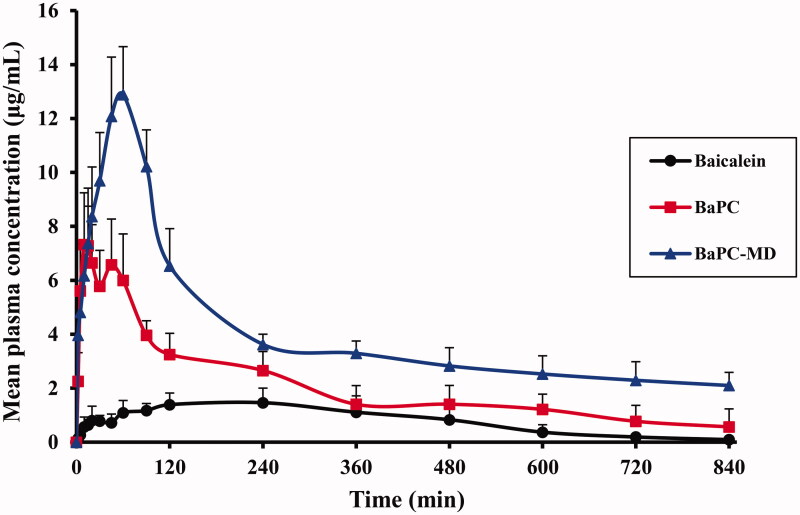
Mean plasma concentration - time curves of baicalin in rats after oral admin-istration of pure baicalein, BaPC and BaPC-MD equivalent to 75 mg/kg of baicalein (*n* = 6). Data were expressed as means ± SD.

Figure 4 displays the mean plasma concentrations of baicalin with time curves after oral administration of pure baicalein, BaPC and BaPC-MD. The mean pharmacokinetic parameters are shown in Table 2. Compared with pure baicalein, the mean value of the *C*_max_ of baicalein after oral administration of BaPC increased from 1.61 to 8.68 μg/mL and the AUC_0–14_ increased from 664.68 to 1748.20 μg/mL min. Statistical analysis showed that there were significant differences in the AUC_0–14 h_ and *C*_max_ between the pure baicalein and BaPC. While, the relative bioavailability of BaPC to pure baicalein was only 263.01%, which was still unsatisfactory and might be ascribed to the low dispersibility of BaPC. When orally administrated BaPC-MD, the average *C*_max_ was 7.92- and 1.47-fold of pure baicalein and BaPC, respectively. In parallel with the *C*_max_, the AUC_0–14 h_ of BaPC-MD enhanced 5.01- and 1.91-fold as great as that of pure baicalein and BaPC, respectively and the differences were of statistical significance. The mean plasma concentration–time curve of BaPC after oral administration exhibited double peaks, which could be attributed to the enterohepatic circulation (Xing et al., 2005). However, the disappearance of double peaks in the mean plasma concentration-time curve of BaPC-MD might be ascribed to the higher plasma concentration than BaPC, which could decrease the influence of enterohepatic circulation on the plasma concentration-time curve of BaPC-MD (Tian et al., 2012).

**Table 2.** Pharmacokinetic parameters of baicalin in rats after oral administration of baicalein, BaPC and BaPC-MD (*n* = 6).

Pharmacokinetic parameters	Baicalein	BaPC	BaPC-MD
*C*_max_ (μg/mL)	1.61 ± 0.37	8.68 ± 1.35*	12.75 ± 1.77* #
*T*_max_ (min)	170.00 ± 65.73	33.33 ± 8.67*	48.33 ± 8.22*
AUC_0–_*_*t*_* (μg/mL min)	664.68 ± 73.50	1748.20 ± 280.80*	3331.04 ± 773.37* #
AUC_0–∞_(μg/mL min)	720.56 ± 283.77	2227.61 ± 753.50*	6218.58 ± 1561.37* #

Data were expressed as means ± SD.

* *p* < 0.05 versus baicalein.

# *p* < 0.05 versus BaPC.

These results indicated that BaPC-MD based on BaPC, using PVP-K30 as matrix carriers, achieved an optimal oral bioavailability. There were several reasons for the excellent results. Firstly, an amorphous state of baicalein in BaPC caused higher aqueous solubility and dissolution of drugs compared to the crystalline state. Secondly, many studies reported that phospholipid complex could also improve the permeability of drugs and thus enhance the oral bioavailability of drugs because of the amphiphilic nature of phospholipids (Saoji et al., 2016). BaPC-MD was prepared by adding PVP-K30 into BaPC in case of not destroying the complexation state of baicalein and phospholipid, which could guarantee the high permeability of BaPC to enhancing drug absorption. Finally, BaPC-MD increased the dissolution of baicalein by improving the low dispersibility of BaPC, and consequently enhanced the oral bioavailability of baicalein on the basis of BaPC.

## Conclusions

In this study, a novel matrix dispersion of baicalein based on phospholipid complex was successfully prepared by a discontinuous solvent evaporation method. Using this approach, baicalein in the BaPC-MD still existed in a complexation state with phospholipids, which guaranteed the high permeability of baicalein. The imperfect dissolution of BaPC was significantly improved by the preparation of BaPC-MD with the matrix carriers of PVP-K30. BaPC-MD delivery system remarkably enhanced the oral bioavailability of baicalein on the basis of BaPC, suggesting that this study came up with a promising strategy to achieve the optimal oral bioavailability for drugs with poor water-soluble drugs.
